# Nutritional value and bioaccumulation of heavy metals in nine commercial fish species from Dachen Fishing Ground, East China Sea

**DOI:** 10.1038/s41598-022-10975-6

**Published:** 2022-04-28

**Authors:** Hong Huang, Yingdong Li, Xinyun Zheng, Zuanyi Wang, Zhenhua Wang, Xiaopeng Cheng

**Affiliations:** 1 College of Marine Ecology and Environment, Shanghai Ocean University, Shanghai, 201306 China; 2 Engineering Technology Research Center of Marine Ranching, Shanghai Ocean University, Shanghai, 201306 China

**Keywords:** Ecology, Environmental sciences

## Abstract

The study evaluated the nutritional quality in muscle tissues of nine commercially important marine fish species. And the concentrations of trace metals (i.e. As, Hg, Cu, Pb, Cr, Cd and Zn) in the muscles (edible part) and tissues (gill and liver) of fishes caught from Dachen fishing ground, the coast of Zhejiang Province, East China Sea, were determined, and the values of target hazard quotient (THQ) and the carcinogenic risk (TR) were calculated for assessing human health risk. Significant differences(P < 0.05) were observed in the proximate chemical composition of fish muscles in these species. The muscle protein content of fish species ranged from 12.36 to 23.41%. The muscle lipid content of fishes ranged from 0.48 to 2.54%. The accumulation capacity of heavy metals (except Cr) in livers and gills was higher than that in muscles. In addition, the accumulation ability of most fishes is related to the water layer they live, the fishes living in the demersal layer showed more accumulation of heavy metals than the middle-upper layer(except Cu). Estimated daily intake (EDI), target hazard quotient (THQ), hazard index (HI) and the carcinogenic risk (TR) assessed for potential human health risk implications suggest that the values were within the acceptable threshold for human. However, the carcinogenic risk(TR) of As and Cr was close to the critical limit (10^–4^). Therefore, in order to ensure the health and safety of human consumption, the continuous monitoring of heavy metals in Dachen fishing ground area is suggested.

## Introduction

In recent decades, the consumption of fish worldwide has been growing rapidly due to their nutritional benefits and high quality proteins^1^. However, heavy metals contamination of fish has caused a great global concern^2^, which also poses a health threat to human health^3,4^. Fish can absorb heavy metals from surrounding water, sediment and their diet^5^, large or improper consumption is likely to cause adverse effects on human body, therefore, it is important and necessary to determine the accumulation of heavy metals contents in the widely consumed economical fish species.

Several methods have been proposed for estimation of the potential risks to human health of heavy metals in fish. Of course, we have never stopped studying this. Among these, carcinogenic and non-carcinogenic effects were extensively used to evaluate the impact on human health. The noncarcinogenic health risk is generally evaluated by estimating target hazard quotient (THQ). The carcinogenic effect is evaluated by providing cancer slope factor for As, Pb and Cd to determine the carcinogenic risk (TR) over a lifetime exposure to As, Pb and Cd. Some studies^6–9^ combined THQ and TR to assess the human health risks associated with consumption fish. These studies and experiments not only provide great help for the follow-up research, but also help to improve people's understanding of health and promote the improvement of human health.

Dachen Islands, known as the Pearl of the East China Sea, which is located in Taizhou Bay, the outer island off the coast of central and southern Zhejiang Province. Dachen Island, with a land area of 14.96 km^2^, is the midpoint of the western Pacific Ocean waterway, also one of the national first-class fishing ports and the second largest fishery in Zhejiang Province. It is an important industry on this island, which is also a tourism spot. Countless people come here and countless related researches are carried out here^10^. Due to unique natural resources and location advantages, there are many economical fish species such as *Larimichthys crocea, Sebastiscus marmoratus*, *Lateolabrax maculatus*, *Muraenesox cinereus*^11,12^. The concentrations of heavy metals in the East China Sea have been widely investigated and reported in previous literatures^13–15^, however, the heavy metal levels in economical fish from Dachen area have not yet been reported. The main purposes of this study are (1) to evaluate the nutritional value of nine commercial marine fish species collected from Dachen fishing ground by determining their proximate composition of protein and lipids; (2) to determine the concentrations of seven heavy metals (Cu, Zn, Pb, Cr, As, Hg, Cd) and compare variations of heavy metal contents and enrichment law in different tissues of fish species from Dachen fishing ground; (3) to conduct the non-carcinogenic and carcinogenic human health risk assessment of the consumption of heavy metals.

## Material and methods

### Study areas and sample collection

**Figure 1:**
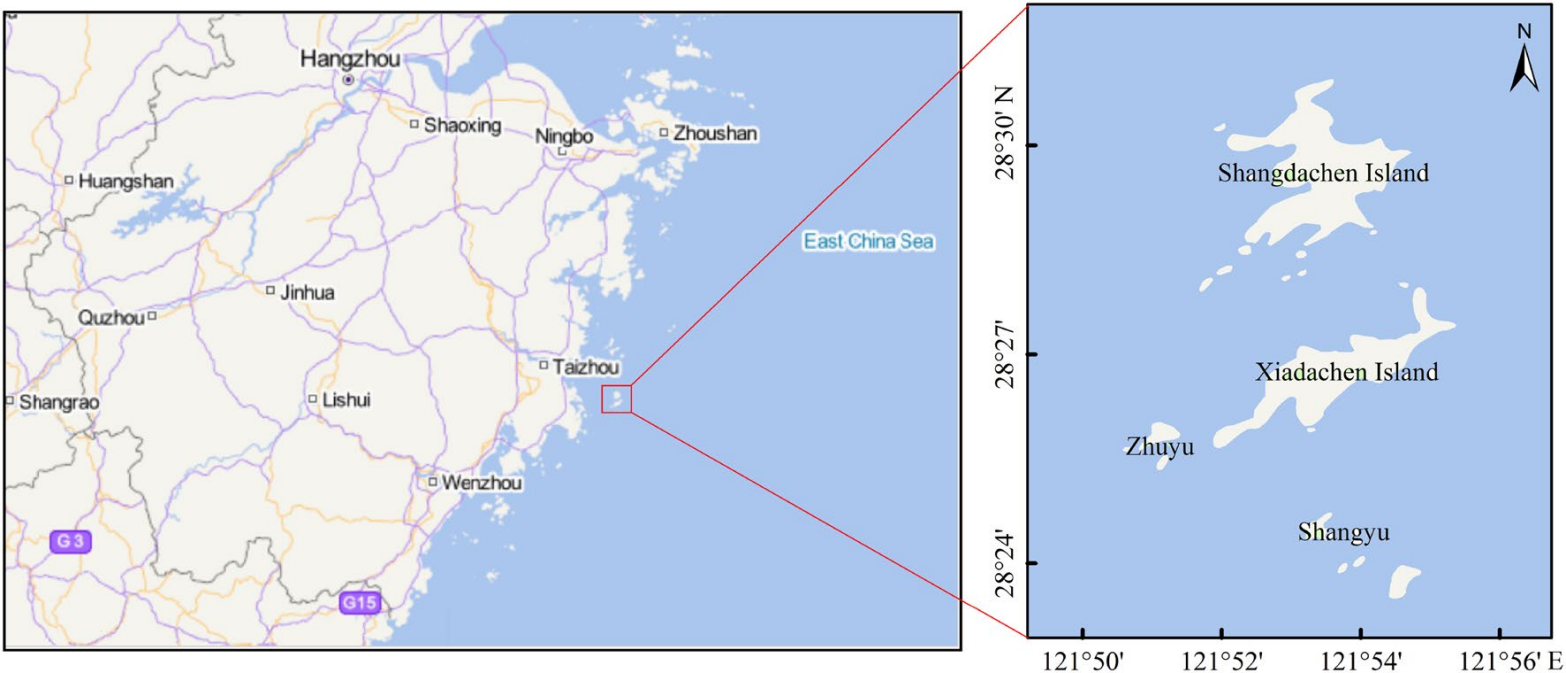
Map of the study area.

**Figure 2:**
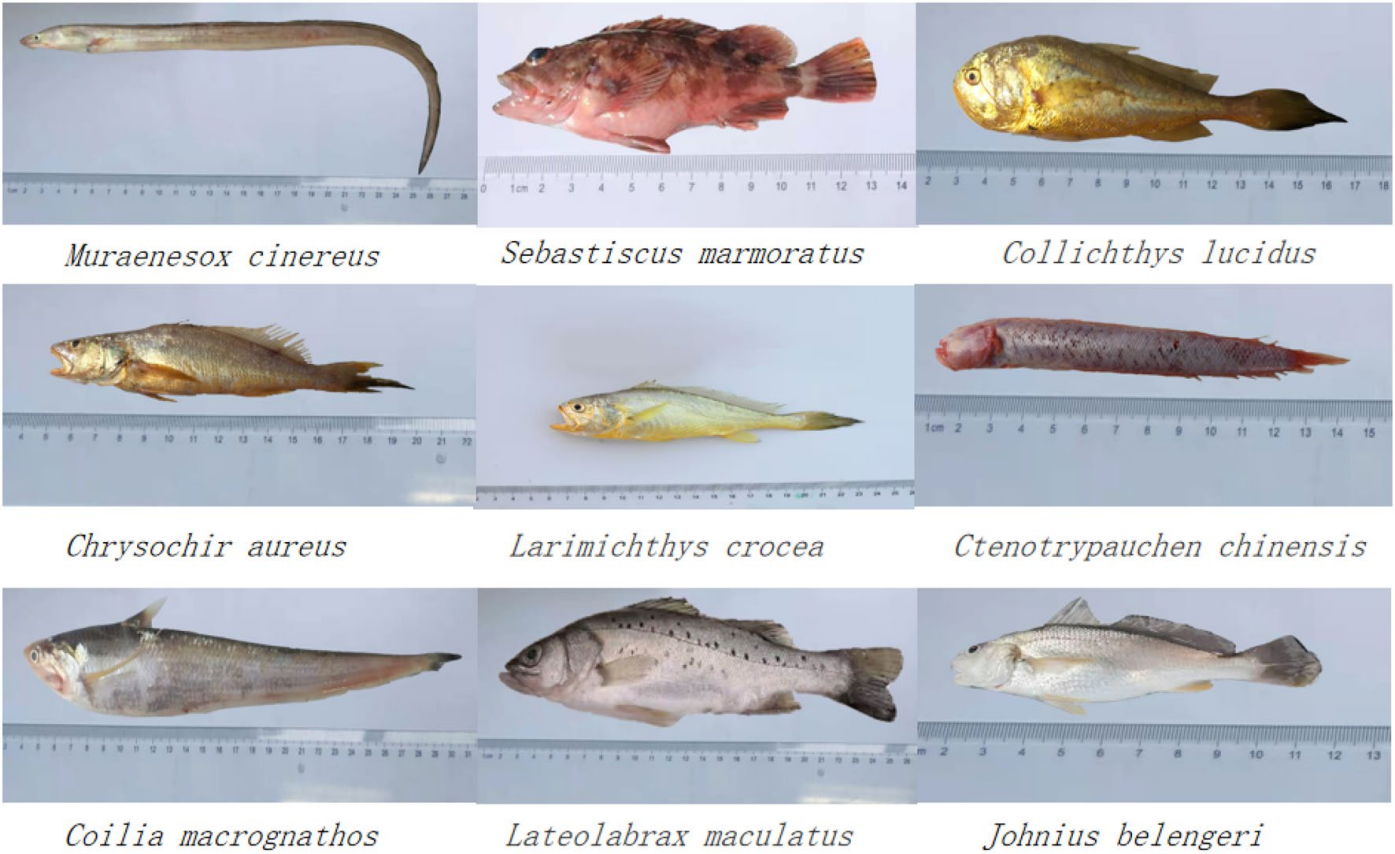
The experimental fish samples collected from Dachen fishing ground, East China Sea.

This study was carried out in the sea area of Dachen Islands (28° 28′ 12.00″ N–28° 22′ 12.00″ N, 121° 48′ 00.00″ E–121° 60′ 00.00″ E) (Fig. 1). The selection of fish species in this paper mainly considered economy and catch. And they represent different water layers. Samples of 9 different fish species were caught by bottom trawling in November, 2019(Fig. 2). Also they are carnivorous and have the potential to bioconcentrate contaminants, which would normally be present in the water or inside sediments. After the fishing ban, the biomass and biodiversity of the fishes are high in the sea area of Dachen Islands. For each species, six samples were collected for this study. The total length and weight for each individual was measured and recorded in Table 1. Sufficient amount of muscle, liver, gills from the fish of the same species were removed from each organism and dissected on-site by clean stainless steel knife. It should be mentioned here that only edible part (fish flesh and skin) were chosen as muscle tissue. Each collected tissue sample was preserved in clean plastic bags and frozen immediately until it was transported to the laboratory. The tissues samples of each individual fish were then air-dried to remove the extra water, and subsequently were dried at − 80 °C for 24 h using a vacuum freeze-drying instrument (Christ Alpha, Germany), rations of moisture were also accurately calculated by comparing the weight before and after drying. Then ground to powder, stored at – 20 °C for succeeding uses.

**Table 1:** Characteristics of fish species from Dachen fishing ground.

Scientific name	Number	Body weight (wet weight, g)	Body length (mm)	Habitat
*Johnius belengeri (JB)*	6	3.75–14.72	70–120	Demersal
*Chrysochir aureus (CA)*	6	5.73–86.43	80–175	Demersal
*Collichthys lucidus (CL)*	6	7.06–28.50	100–153	Demersal
*Ctenotrypauchen chinensis (CC)*	6	8.19–16.49	90–160	Demersal
*Muraenesox cinereus (MC)*	6	11.92–550.50	246–692	Demersal
*Sebastiscus marmoratus (SM)*	6	23.91–52.59	111–147	Demersal
*Coilia macrognathos (CM)*	6	37.00–96.63	245–319	Middle-upper
*Larimichthys crocea (LC)*	6	52.50–84.20	180–204	Demersal
*Lateolabrax maculatus (LM)*	6	97.47–141.24	226–285	Middle-upper

*ww *wet weight.

Seawater samples for heavy metal determination were collected in acid washed polyethylene bottles. The bottles were rinsed thoroughly with deionised water after being washed in dilute nitric acid (HNO_3_). In the field the bottles were rinsed several times with the seawater and 1 L of water sample was then collected at about 50 cm below the water surface. And seawater samples were filtered through Whatman No. 45 filter paper. The seawater samples were acidified with concentrated nitric acid for preservation. The acid pretreatment ensured that heavy metals did not get absorbed to the surface of the container during transportation and storage.

A total of 10 surface sediment samples were collected at sampling sites distributed throughout the study area (Fig. 1). A stainless-steel grab sampler was used for collection, while a plastic shovel was used to excavate the sediments from the middle part of the sampler. Samples were kept in pre-cleaned polyethylene bags and frozen until lab analysis.

### Sample analysis

The proximate composition of fish muscles (protein and lipids) was determined according to the standard methods of AOAC^16^. The metal extraction procedure is referred to a previous study^17^ and we have made corresponding improvements based on this research. Both fish and sediment samples were weighed in duplicate. For fish and sediment samples, about 0.5 g of each powdered sample was digested in microwave digester (MDS-6G, China) using the ratio of 2:1 (8 mL + 4 mL) of concentrated nitric acid (GR) and hydrogen peroxide (AR). After digestion process, one of them does not removed acid to determine arsenic and mercury. And the other transferred the sample to a 30 mL polytetrafluoroethylene beakers and heated it on hot plate at 170 °C until about 1 mL of solvent remains to determine other heavy metals. Then samples were diluted with deionized water in 25 mL polypropylene tubes. The concentration levels of Cu, Zn, Pb, Cd and Cr in the digested sample solutions were determined by using inductively coupled plasma optical emission spectrometer (ICP-OES, Optima8000, USA), and the Hg and As contents were determined by using atomic fluorescence spectrometry (AFS-3100, China). Certified Reference Material (Standard oyster Tissue 1566a) obtained from the U. S. Department of Commerce was also analyzed routinely every 10 samples and all runs were analyzed in triplicated as quality control. In addition, reagent blanks were analyzed to provide a baseline correction for the results of the samples. The mean recoveries of metals were between 91.8 and 105.2%, indicating a decent agreement between certified and measured values.

For seawater sample, detailed operating procedures are described in the Specifcation for Marine Monitoring of China (SMMC) (GB17378.4-2007, ICS 07.060 A 45)^18^.

### Statistical analyses

Data were given as mean ± SD for each of the measured variables. All statistical analyzes were performed using SPSS version 21.0 version. The one-sample Kolmogorov–Smirnov (K–S) test was used to assess the data normality. All the concentration values for seven heavy metals in the tissues of fish species were normally distributed at the 95% confidence level. Therefore, a one-way ANOVA test was used to assess significant differences tissues. Turkey's Post hoc test was employed to test the significance of difference between single species in different organ.

### Health risk assessment

Several methods have been proposed for estimating the potential risks to human health of heavy metals in fishes^19–21^. These methods were described in detail below. Mean concentrations of heavy metals for each species were used in all calculations.

#### Estimated daily intake (EDI)

Health risk was estimated considering the average concentrations of all fish muscles and daily heavy metal intake (EDI). The specific formula is as follows^9^:

1 $$ EDI = \frac{EF \times ED \times FIR \times C}{{BW \times AT}} \times 10^{ - 3} \begin{array}{*{20}c} {} & {(1)} & {} & {} \\ \end{array} $$

where C is the concentration of heavy metals in the selected fish tissues (mg/kg, ww); FIR is the food ingestion rate, which is 31 g/person/day^22^; ED is the exposure duration 70 years^23^; EF is the frequency of exposure 365 days/year^23^; BW is the average body weight 55.9 kg^24^; AT is the average exposure time 25,500 days^9^.

#### Non-carcinogenic health risk

The target hazard quotient (THQ) and hazard index (HI) are a method proposed by the U. S. Environmental Protection Agency (USEPA) for assessing the risk of heavy metals caused by food intake by the human body^23^. The value of ratio < 1 implies a non-significant risk effects. The specific formula is as follows^23^:

2 $$ THQ = \frac{EF \times ED \times FIR \times C}{{RFD \times BW \times AT}} \times 10^{ - 3} \begin{array}{*{20}c} {} & {(2)} & {} & {} & {} \\ \end{array} $$

3 $$ HI = \sum {THQ\begin{array}{*{20}c} {} & {(3)} & {} & {} \\ \end{array} } $$

RFDs of the different heavy metals for example As, Hg, Cd, Cu, Zn, Pb, and Cr are 0.0015, 0.00016, 0.001, 0.04, 0.3, 0.004 and 1.5, repectively^23^. Other factors have been mentioned as above.

#### The carcinogenic risk (TR)

As well as non-carcinogenic risks, there are also carcinogenic risks in human health risk assessment^17^. All trace metals do not have carcinogenic effects. However, As, Pb, Cd and Cr among the studied heavy metals are considered as carcinogens. For carcinogens, the individual risk assessment increases the probability of developing cancer due to exposure to potential carcinogens^22,25^. The acceptable risk levels of TR^5,9^ for carcinogens ranged from 10^–6^ to 10^–4^. The model formula is as follows^26^:

4 $$ TR = \frac{EF \times ED \times FIR \times C \times CSFo}{{BW \times AT}} \times 10^{ - 3} \begin{array}{*{20}c} {} & {(4)} & {} \\ \end{array} $$

where the oral carcinogenic slope factor (CSFo) was obtained from the database of the U. S. Environmental Protection Agency^26^. Available CSFo values (mg/kg/day) are: As (1.5), Pb (0.0085), Cd (6.3) and Cr (0.5)^26^. Other factors have been mentioned as above. Assume that 10% of the total As can be assessed as inorganic state^5,27,28^ in this study.

### Ethical approval

Fish used for this study were fresh but lifeless, however all procedures used conform to standard scientific research guidelines. All methods are reported in accordance with ARRIVE guidelines. All experimental protocols were approved by the Shanghai Ocean University. The study was approved by the ethic committee of the Shanghai Ocean University.

## Results

### Chemical composition of fish muscles

The chemical composition of fish muscles is shown in Table 2. The muscle protein content in *Larimichthys crocea* (23.41%) was significantly higher than that in the other species, followed by *Muraenesox cinereus* (21.28%) whereas the muscle protein content in *Ctenotrypauchen chinensis* (12.36%) was the lowest. The muscle lipid content of *Sebastiscus marmoratus* had significantly higher lipid content (2.54%), while *Ctenotrypauchen chinensis* had significantly lower lipid content (0.48%). And compared with other fishing grounds, the muscle protein content of fishes basically was the same (Table 2). The muscle lipid content of fishes ranged from 0.48 to 2.54% and lower than the reported levels in other fishing Ground (Table 2).

**Table 2:** The chemical in the fish muscles of nine fish species collected from Dachen fishing ground.

Scientific name	Site	Protein (%)	Lipid (%)
*Chrysochir aureus (CA)*	This study	19.78 ± 0.42^a^	0.54 ± 0.26^a^
*Larimichthys crocea (LC)*	This study	23.41 ± 0.63^c^	1.16 ± 0.34^a^
	Zhoushan Fishing Ground^29^	21.46–23.80	4.34–9.76
*Collichthys lucidus (CL)*	This study	14.42 ± 0.29^b^	2.00 ± 0.71^b^
	Zhoushan Fishing Ground^30^	14.47–15.33	3.45–3.95
*Ctenotrypauchen chinensis (CC)*	This study	12.36 ± 0.31^b^	0.48 ± 0.13^a^
	Bohai Fishing Ground^31^	11.66–13.80	2.52–2.54
*Coilia macrognathos (CM)*	This study	18.70 ± 0.48^b^	1.45 ± 0.39^c^
	Bohai Fishing Ground^32^	18.11–18.81	5.25–5.37
*Muraenesox cinereus (MC)*	This study	21.28 ± 0.51^c^	1.16 ± 0.29^c^
	Southern Coastal Fishing Ground^33^	19.60	2.0
*Johnius belengeri (JB)*	This study	15.38 ± 0.19^a^	0.52 ± 0.12^a^
	Southern Coastal Fishing Ground^34^	16.92	7.19
*Sebastiscus marmoratus (SM)*	This study	19.05 ± 0.28^c^	2.54 ± 0.77^b^
	Zhoushan Fishing Ground^30^	17.90 ± 0.43	3.70 ± 0.25
*Lateolabrax maculatus (LM)*	This study	15.87 ± 0.22^a^	1.76 ± 0.67^c^
	Bohai Fishing Ground^35^	16.51 ± 0.12	1.95 ± 0.08

Values in the same column having different superscript letters are significantly different(P < 0.05).

### Heavy metals in fish samples

#### The content of heavy metals in fish muscles

**Figure 3:**
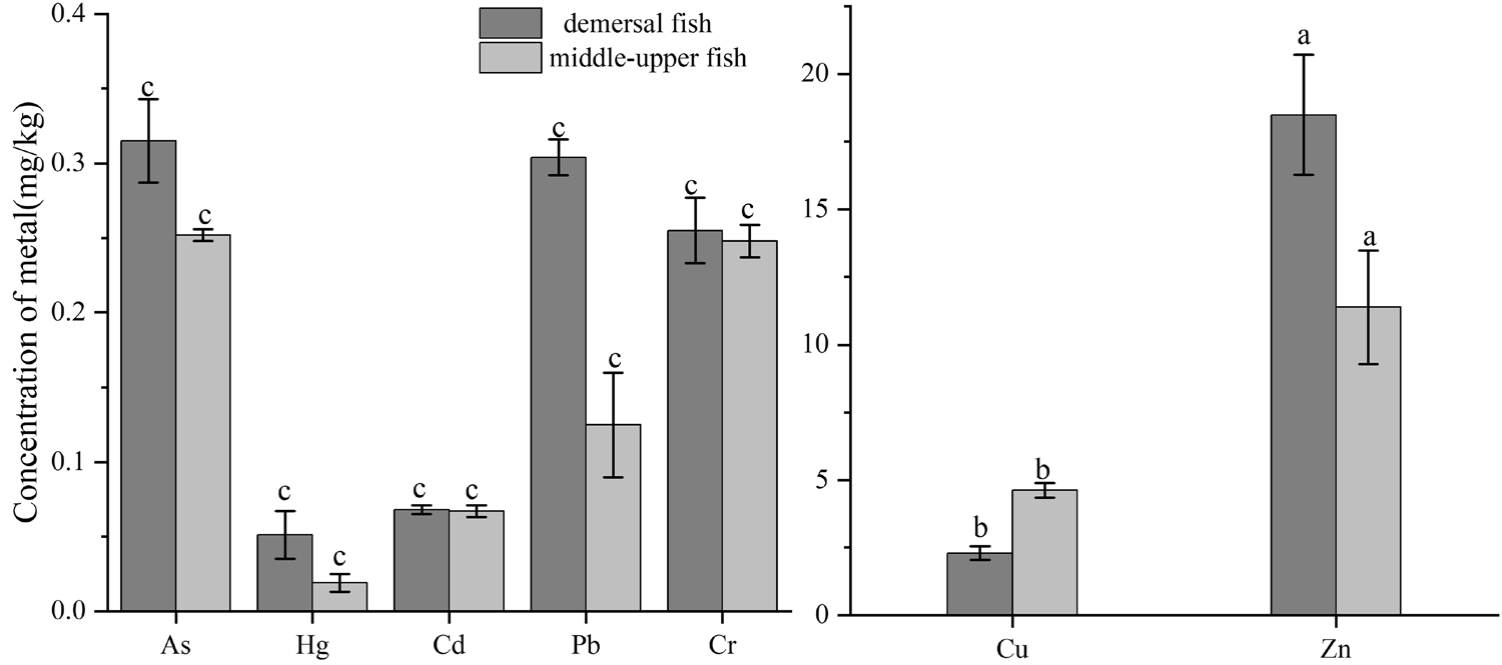
Distribution of heavy metals in different habitats. Different letters are significantly different (P < 0.05).

In the present study, the concentrations of seven heavy metals (As, Hg, Cd, Cu, Zn, Pb, and Cr) in the muscles of nine fish species were shown in Table 3. Metal concentrations were reported using wet-weight. The concentration of all the examine heavy metals in fish muscles remained well below the acceptable limits for human consumption established by FAO. The order of mean concentrations of heavy metals from high to low was shown as follows: Zn (16.910 mg/kg) > Cu (2.810 mg/kg) > As (0.301 mg/kg) > Pb (0.264 mg/kg) > Cr (0.074 mg/kg) > Cd (0.067 mg/kg) > Hg (0.044 mg/kg). It was found that the accumulating capacity of most fishes was linked to the water layer in which they lived (Fig. 3). The results obtained from this study were analyzed using the analysis of variance (ANOVA), and the differences between essential (Cu and Zn) and non essential metals (As, Hg, Cd, Pb and Cr) concentration levels were considered significant at 95% confidence interval (P < 0.05). The amount of heavy metals in the demersal fish was greater than the middle-upper fish (except Cu).

**Table 3:** Concentration of heavy metals of fish and marine environment.

Species	Tissues	Heavy metals (mg/kg, ww)						
		As	Hg	Cd	Cu	Zn	Pb	Cr
LC	Muscle	0.140 ± 0.014	0.026 ± 0.005	0.067 ± 0.006	2.000 ± 0.028	12.550 ± 0.806	0.025 ± 0.003	0.036 ± 0.006
	Gill	0.250 ± 0.085	0.014 ± 0.004	0.067 ± 0.001	3.300 ± 0.055	52.400 ± 3.408	0.350 ± 0.028	0.234 ± 0.015
	Liver	0.165 ± 0.010	0.023 ± 0.004	0.096 ± 0.007	15.900 ± 1.400	59.950 ± 1.838	0.100 ± 0.028	0.270 ± 0.014
MC	Muscle	0.412 ± 0.014	0.066 ± 0.009	0.068 ± 0.007	2.300 ± 0.283	21.400 ± 1.853	0.100 ± 0.014	0.075 ± 0.008
	Gill	0.220 ± 0.091	0.023 ± 0.006	0.069 ± 0.003	7.300 ± 0.360	47.800 ± 2.687	0.050 ± 0.001	0.253 ± 0.003
	Liver	0.802 ± 0.127	0.036 ± 0.003	0.096 ± 0.007	36.902 ± 4.667	77.410 ± 1.754	0.150 ± 0.016	0.260 ± 0.001
SM	Muscle	0.245 ± 0.033	0.111 ± 0.018	0.067 ± 0.010	2.400 ± 0.141	27.150 ± 2.150	1.100 ± 0.042	0.092 ± 0.006
	Gill	0.350 ± 0.085	0.009 ± 0.001	0.066 ± 0.011	1.700 ± 0.905	49.400 ± 1.131	0.050 ± 0.003	0.251 ± 0.004
	Liver	0.285 ± 0.016	0.037 ± 0.006	0.072 ± 0.006	11.950 ± 1.372	70.502 ± 2.192	0.051 ± 0.006	0.251 ± 0.003
JB	Muscle	0.255 ± 0.013	0.037 ± 0.017	0.066 ± 0.004	1.300 ± 0.127	6.250 ± 0.311	0.650 ± 0.014	0.089 ± 0.011
	Gill	0.271 ± 0.072	0.105 ± 0.008	0.089 ± 0.003	6.452 ± 0.170	34.900 ± 0.311	0.110 ± 0.014	0.207 ± 0.004
	Liver	0.255 ± 0.010	0.006 ± 0.002	0.067 ± 0.011	2.900 ± 0.156	74.804 ± 0.580	1.850 ± 0.014	0.198 ± 0.003
CA	Muscle	0.430 ± 0.042	0.027 ± 0.011	0.067 ± 0.001	3.050 ± 0.099	17.300 ± 0.580	0.050 ± 0.003	0.104 ± 0.008
	Gill	0.215 ± 0.083	0.021 ± 0.001	0.068 ± 0.004	3.550 ± 0.339	50.702 ± 2.418	0.100 ± 0.014	0.184 ± 0.003
	Liver	0.470 ± 0.071	0.038 ± 0.010	0.072 ± 0.004	9.352 ± 0.509	67.051 ± 1.470	0.102 ± 0.018	0.249 ± 0.003
CC	Muscle	0.490 ± 0.099	0.082 ± 0.018	0.067 ± 0.003	2.200 ± 0.240	29.600 ± 3.309	0.100 ± 0.001	0.074 ± 0.001
	Gill	–	–	–	–	–	–	–
	Liver	–	–	–	–	–	–	–
CL	Muscle	0.235 ± 0.057	0.010 ± 0.004	0.067 ± 0.008	2.800 ± 0.283	15.150 ± 0.226	0.100 ± 0.016	0.065 ± 0.001
	Gill	0.215 ± 0.071	0.009 ± 0.002	0.068 ± 0.003	3.752 ± 0.269	73.15 ± 2.546	0.150 ± 0.002	0.233 ± 0.004
	Liver	0.685 ± 0.033	0.018 ± 0.002	0.082 ± 0.011	9.306 ± 0.552	58.750 ± 1.428	0.053 ± 0.003	0.217 ± 0.004
CM	Muscle	0.249 ± 0.018	0.023 ± 0.005	0.067 ± 0.007	4.160 ± 0.198	9.900 ± 0.283	0.150 ± 0.017	0.061 ± 0.006
	Gill	0.350 ± 0.156	0.031 ± 0.001	0.069 ± 0.007	3.553 ± 0.297	13.600 ± 0.707	0.202 ± 0.014	0.267 ± 0.004
	Liver	0.455 ± 0.044	0.077 ± 0.002	0.092 ± 0.014	9.350 ± 0.156	20.902 ± 1.612	0.052 ± 0.004	0.249 ± 0.002
LM	Muscle	0.255 ± 0.034	0.015 ± 0.005	0.067 ± 0.001	5.100 ± 0.707	12.85 ± 0.269	0.100 ± 0.003	0.076 ± 0.003
	Gill	0.235 ± 0.048	0.048 ± 0.011	0.068 ± 0.007	2.850 ± 0.283	24.150 ± 1.216	0.352 ± 0.014	0.228 ± 0.003
	Liver	0.090 ± 0.012	0.006 ± 0.001	0.067 ± 0.001	2.602 ± 0.424	13.600 ± 1.414	0.360 ± 0.016	0.201 ± 0.001
Water(mg/l) × 10^–3^		1.28 ± 0.62	0.031 ± 0.007	0.051 ± 0.015	3.15 ± 1.50	15.67 ± 10.70	3.28 ± 1.89	0.84 ± 0.34
Sediment		1.488 ± 0.980	0.029 ± 0.021	0.079 ± 0.015	28.27 ± 5.52	73.05 ± 7.65	19.95 ± 3.98	54.47 ± 15.21
FAO^36^		1.0	1.0	0.2	10	30	2.5	1.0

The gills and liver of the CC were not sampled.

The levels indicated by the FAO are for fish.

#### Distribution of heavy metals in different tissues of fish

**Figure 4:**
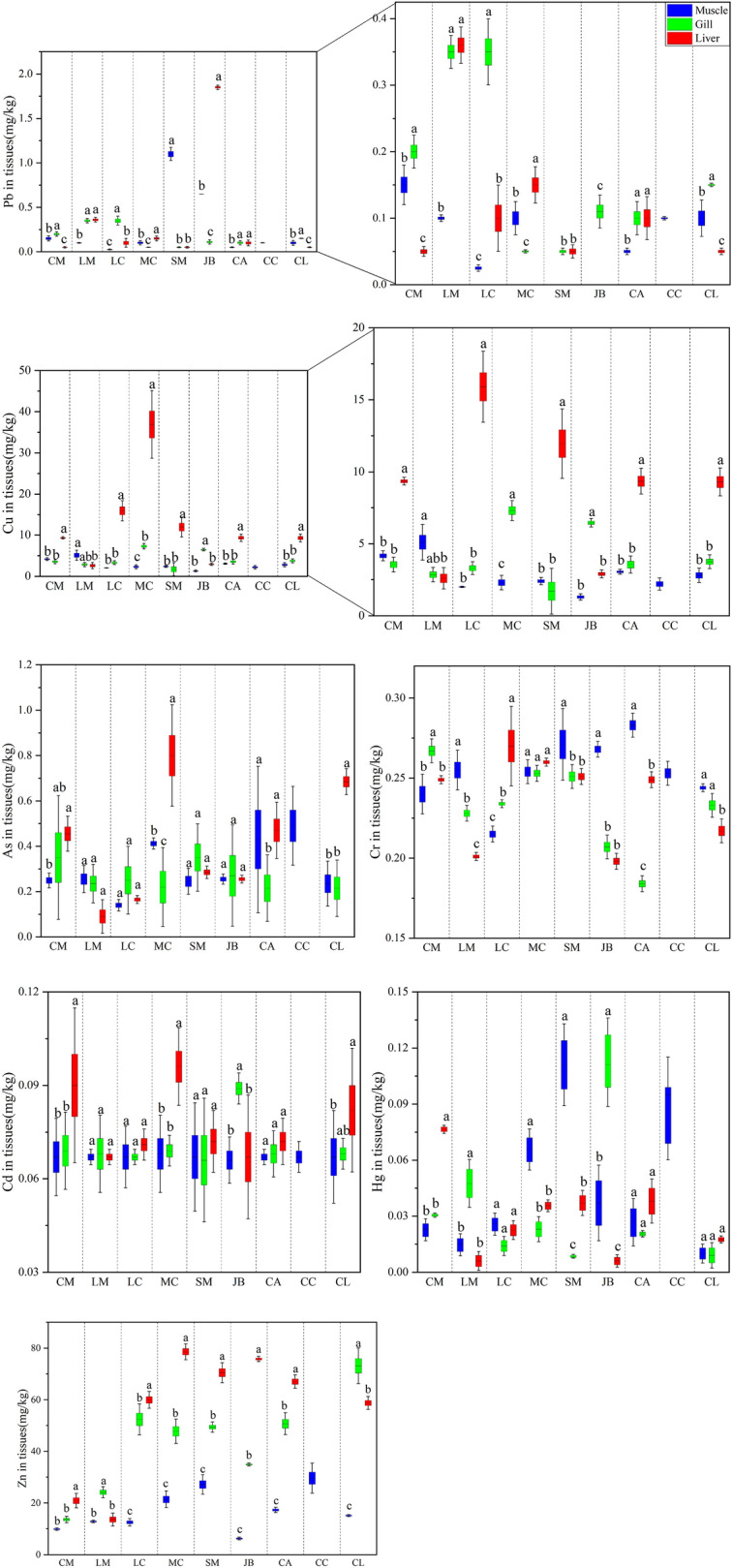
Concentrations of heavy metals (ww) in the muscles (blue), gills (green), and liver (red) of nine fish species collected from Dachen fishing ground, East China Sea. Different letters are significantly different within the same fish (P < 0.05).

The concentrations of seven heavy metals in various tissues of nine marine fish species were shown in Fig. 4. In this study, the accumulation of heavy metals in most fish was higher in liver or gills than in muscle. The content of Zn in the liver of *Muraenesox cinereus* was the highest (77.410 mg/kg). And the content of Zn in muscle of *Muraenesox cinereus* was also relatively high. Moreover, the content of Hg in the liver of *Johnius belengeri* was the lowest (0.006 mg/kg). The accumulation ability of heavy metals varies among different tissues in the same fish. Our data indicated that the mean concentrations of heavy metals in muscle, liver and gills differed significantly (P < 0.05) in most fish species. The heavy metal Cu content in the liver and gill of *Muraenesox cinereus* exceeded that of the muscle. The accumulation ability of Cu in other fish was similar to *Muraenesox cinereus*. However, based on our results, it was found that the ability to accumulate Cr was different from that of Cu. Other heavy metals have the same accumulation capacity as Cu.

### Health risk assessment

#### Estimated daily intake (EDI)

The EDI values of seven heavy metals calculated for nine fish species were given in Table 4. The EDI value of Zn in all fish species were found higher, while those of Hg in most fish species were found lower. The EDI value of seven heavy metals calculated for each fish species was very lower than the tolerable daily intake (TDI) value, which suggested that the daily intake of seven heavy metals via the ingestion of nine fish species in this study would pose no detrimental health risk to humans.

**Table 4:** Estimated daily intake (EDI) established for the consumption of fish from Dachen fishing ground, East China Sea (units ug/kg bw/day for EDI and TDI, ww).

Species	EDI						
	As	Hg	Cd	Cu	Zn	Pb	Cr
LC	0.0233	0.0141	0.0372	1.1091	6.9597	0.0139	0.1192
CM	0.0415	0.0125	0.0372	2.3070	5.4902	0.0832	0.1331
CL	0.0391	0.0055	0.0372	1.5528	8.4016	0.0555	0.1353
MC	0.0686	0.0363	0.0377	1.2755	11.8676	0.0555	0.1409
SM	0.0408	0.0616	0.0372	1.3309	15.0564	0.6100	0.1503
LM	0.0425	0.0080	0.0372	2.8283	7.1261	0.0555	0.1414
CA	0.0716	0.0147	0.0372	1.6914	9.5939	0.0277	0.1569
JB	0.0425	0.0205	0.0366	0.7209	3.4660	0.3605	0.1486
CC	0.0816	0.0455	0.0372	1.2200	16.4150	0.0555	0.1403
TDI	2.14^37^	0.08^38^	0.8^39^	500^40^	300^40^	1.5^41^	300^42^

#### Non-carcinogenic health risk

The estimated target hazard quotient (THQ) for individual heavy metal from the consumption of various fish species was shown in Table 5. The THQ results for seven heavy metals in nine marine fishes revealed that THQ values for all metal in nine fishes were below one, and the THQ values of different heavy metal of the same species were quite different. And, hazard index (HI) values of combined heavy metals calculated for each fish species were below one. The THQ and HI results suggested that non-carcinogenic health risk from the intake of individual or combined heavy metals in the fish species were not expected for consumers.

**Table 5:** The target quotients(THQ) and hazard index(HI) established for the consumption of fish from Dachen fishing ground, East China Sea(ww).

Species	THQ							HI
	As	Hg	Cd	Cu	Zn	Pb	Cr	
LC	0.0776	0.0884	0.0372	0.0277	0.0232	0.0035	8.93E−05	0.2576
CM	0.1381	0.0780	0.0372	0.0577	0.0183	0.0208	9.82E−05	0.3501
CL	0.1303	0.0347	0.0372	0.0388	0.0280	0.0139	1.01E−04	0.2829
MC	0.2285	0.2270	0.0377	0.0319	0.0396	0.0139	7.90E−05	0.5786
SM	0.1359	0.3847	0.0372	0.0333	0.0502	0.1525	1.02E−04	0.7938
LM	0.1414	0.0503	0.0372	0.0707	0.0238	0.0139	7.59E−05	0.3372
CA	0.2385	0.0918	0.0372	0.0423	0.0320	0.0069	1.19E−04	0.4488
JB	0.1414	0.1282	0.0366	0.0180	0.0116	0.0901	1.07E−04	0.4260
CC	0.2717	0.2842	0.0372	0.0305	0.0547	0.0139	8.24E−05	0.6922

#### The carcinogenic risk (TR)

Due to the high toxicity of As, Pb, Cd and Cr the target cancer risk (TR) for these four heavy metal elements was estimated and the results were shown in Table 6. The measured TR values of As, Pb and Cd were ranged from (2.33–8.15) × 10^–5^, (0.01–0.52) × 10^–5^, (4.68–4.75) × 10^–5^ and (5.96–7.85) × 10^–5^, respectively. The results showed calculated TR values were within the risk as acceptable range is 10^–6^ to 10^–4^. From the point of As, Pb, Cd and Cr TR values, the fish will not cause carcinogenic effects on humans through food consumption. Among them, two fishes (*Sebastiscus marmoratus* and *Johnius belengeri*) were higher TR values of Pb than other fishes, which should be given a great concern.

**Table 6:** TR values of As, Pb, Cd and Cr for different species.

Species	As (× 10^–5^)	Pb (× 10^–5^)	Cd (× 10^–5^)	Cr (× 10^–5^)
LC	2.33	0.01	4.68	5.96
CM	5.41	0.07	4.68	6.66
CL	3.91	0.05	4.68	6.77
MC	6.92	0.05	4.75	7.04
SM	4.08	0.52	4.68	7.51
LM	4.24	0.05	4.68	7.07
CA	7.15	0.02	4.68	7.85
JB	4.24	0.31	4.61	7.43
CC	8.15	0.05	4.68	7.02

## Discussion

In this study, the amount of muscle protein in fish species ranged from 12.36 to 23.41%. The study reported that the higher protein content observed in the fish was due to its feeding habits^43^. The protein contents of varies fish were found to be possibly due to their food availability^44^. And no correlation was observed between protein contents and heavy metals in fish muscle (using Pearson correlation test, P < 0.05). Studies^45^ also had shown that there was no correlation between heavy metals and protein content in muscle tissue. This is consistent with our study. And the muscle lipid content of fish ranged from 0.48 to 2.54%, where it varied significantly. Younis et al.^46^ stated lipid content is affected by feeding habits and the territorial food. Nath et al.^47^ reported that lipid content is influenced by the life cycle and environment. In the present study, no correlation was observed between lipid contents and heavy metals in fish muscle (P < 0.05). The differences in the chemical of marine fishes in this study may be due to difference in species, feeding habits, and this agrees with the study^46,48^.

This study found that the content of essential micronutrients (Zn, Cu) is higher than that of non-essential micronutrients (Pb, Cd, As, Hg, Cr). It might because the automatic adsorption of Zn and Cu is processed by organisms, which causes the content of Zn and Cu in fish muscles is greater than that of Pb, Cd, As, Hg and Cr. Zn is a component of a variety of enzymes in the body, and Cu is a component of various oxides in the body^49^.

The amount of heavy metals in the demersal fish was greater than the middle-upper fish (except Cu). This was consistent with the research by Sun et al.^50^. Demersal fish were easily exposed to heavy metals from sediments, which were seen as the main source of heavy metals in marine fish^51,52^. The content of Hg in fish muscle living in the demersal was significantly higher than that of the middle-upper layer. UNEP^53^ research showed that marine fish mercury is methyl mercury, mainly from the marine environment and the food chain transmission. Methyl mercury is primarily caused by the biological and abiotic methylation process of inorganic mercury^54^. Especially, the process of mercury bioremediation on the surface sediment might resulted in the bottom seawater with higher levels of methyl mercury. The corresponding mercury content of demersal fish was greater than that of middle-upper fish. The mercury content of heavy metals in water and sediment was simultaneously detected. The results suggested the mercury content in the sediment was higher than that in the seawater. However, Cu in demersal fish was lower than that in the middle-upper. The research by Yang et al.^55^ had also similar founding, the bioavailability of heavy metals in fish with varied habitats being different.

The accumulation ability of heavy metals in different tissues of the same fish was different. Our data indicated that the mean concentrations of heavy metals in muscle, liver and gills differed significantly (P < 0.05) in most fish species. Different fish have different ability to accumulate heavy metals. This may be linked to the lifespan of fish in the water body and the difference in physiological metabolism^56^. This was in line with the research by Monroy et al.^57^ on the concentration and distribution of heavy metals in fish. It indicated that the liver and gills were the main organs for heavy metal accumulation in fish. The unique structure of the gills is conducive to the penetration of ions in the water, making the gills the central part of fish to directly absorb heavy metals from the water environment^58,59^. The enrichment of heavy metals in the liver was mainly associated with the induction and bonding of metallothionein. The liver is a tissue that continually accumulates, biotransforms, and detoxifies. Therefore, measuring the content of heavy metals in the liver was beneficial to assess the level of heavy metal pollutants in the environment^60,61^. This demonstrated that the primary means of heavy metals entering fish include food intake, branchial membrane adsorption, liver digestion and absorption, and accumulation. However, based on our results, it was found that the ability to accumulate Cr was different from that of Cu. Generally, the content of Cr in muscle was higher than that of the gills and the liver. Since the low absorption rate and a relatively high excretion rate of Cr, the retention rate of Cr in fish is low^62^, resulting the reduction of Cr to some extent in the liver and gills.

Because the content of heavy metal As in marine organisms is generally relatively high, in marine organisms, arsenic occurs mainly in the fasts of organic compounds, as arsenobetaine^27^. Its toxicity is expected to be very low, and it can be quickly eliminated from the body after swallowing. The toxic inorganic arsenic generally only accounts for 0.1–10% of the total arsenic content^27^. Assume that the maximum rate of 10% was used to estimate the level of inorganic arsenic content in fish in the Dachen Sea. In this case, the As content of all the samples in this study was comparatively low. The source of arsenic pollution may be domestic sewage and the use of arsenic-containing pesticides^63^. However, mean arsenic content (0.301 mg/kg) of this study differs from that of Peng et al.^14^ (1.600 μg/g, total arsenic). Future studies should address the As forms accumulated in the edible muscle.

Moreover, Pb is toxic to organisms, can disrupt normal metabolic activities, transmitted through the food chain, enriched and accumulated, and can be transformed into more toxic organic compounds under certain conditions^64^. Lee et al.^65^ had found that long-term exposure to high levels of Pb can damage the brain, liver and kidneys and even reduce the function of the nervous system, eventually leading to death. This study found that the enrichment trend of Pb was similar to that of Cu, and its content in the gills and liver was higher than that muscle. The average content of Pb (0.264 mg/kg) in this study was consistent with that of Li^66^ (0.128 mg/kg) but different from that of Peng et al.^14^ (5.700 mg/kg). It may be caused by the development of coastal industries in Zhejiang province over the past few years. The concentration of Pb in the aquatic environment is higher, which exceeds the fish body’s discharge capacity, resulting in a parallel accumulation of Pb in the fish body (gills, liver, muscle). Studies^63,67^ have reported that the industrial wastewater and domestic sewage generated by the pharmaceutical, chemical and electronic electroplating industries in the Jiaojiang Estuary water were directly discharged into the water, resulting in high lead content. Dachen Island is located in the southeast of Jiaojiang River estuaries and is highly susceptible to lead pollution from the Jiaojiang River estuaries. Besides, due to the development and utilization of fishing and ship transportation in the Dachen sea area in recent years, the wastewater produced by the local maritime transport has also caused lead pollution to a certain extent.

The heavy metal pollution is one of important environmental factors that can considerably affect human health. The muscles of fish may enter into human metabolism through food consumption, leading to serious health risks^68^. Therefore, this study assessed the impact on human health by studying heavy metal pollution in fish in the Dachen Island region. Moreover, it is also conducive to the construction and management of marine ranches^10^.

The EDI reflects the daily exposure to the heavy metal, and is executed to avoid any harmful effect on human health^28^. The EDI values lower than TDI guidelines suggested a lower possible health risk of the heavy metals to the consumers. However, it would not be wise to take it as a permanent measurement to reach a final conclusion^28,69^.

The THQ and HI values below one. There was no potential non-carcinogenic effect for the consumers due to intake of the fish species. Studies carried out by several authors in similar conditions were in line with our results^7,9,28^. However, due to multiple simultaneous pollutants, human could dramatically suffer in the long run^70^. The THQ values of different heavy metal of the same species were quite different. It was found that the risk of Cr was relatively low. Many kinds of literature^71,72^ have suggested that the potential risk of Cr was low, which was also proved by this study, and the potential health risk of Cr was the lowest, which may be ascribed to its higher RFD value.

The results showed calculated TR values were within the risk as acceptable range is 10^–6^ to 10^–4^, and consumers were less prone to carcinogenic. In fact, 90% of the carcinogenic risk is observed in the As contaminated acquatic food items^28^. The inorganic form of As is more lethal than organic one^27,73,74^, and only 10% of the total As can be assessed as inorganic state^5,28^. In this study, although the TR value of As was within an acceptable range, regular monitoring of fish in this fishery was still essential.

In this study, we assumed that the intake of heavy metals was equivalent to the absorption of heavy metals, without considering the time and residual content of heavy metals in the human body. This hypothesis improves the calculation of carcinogen risk. Some heavy metals may be excreted. The heavy metals levels in blood, and urine are suggested to be measured in the future.

## Conclusion

In this study, the results showed that all nine fish species were a good source of protein and lipids. The concentrations of seven heavy metals in various tissues of nine marine fish species were compared and found to be varied considerably among tissues and species. Maximum and minimum of heavy metals concentrations in the fish respectively were determined as Zn and Hg. The accumulation capacity of heavy metal (except Cr) in the liver and gills was higher than that of muscle. The cumulative capacity of heavy metals in fish muscle was linked to the water layer they live, that was, the demersal fish contained more heavy metal than the middle-upper ones (except Cu). From the perspective of human health, the EDI of each element was lower than the respective recommended tolerable daily intake. The THQ and HI values of fish indicated that fish in the Dachen fishing ground were safe to eat. From the point of As, Pb, Cd and Cr TR values, the fish may not cause carcinogenic effects on humans through food consumption. However, the carcinogenic risk (TR) of As and Cr was close to the critical limit (10^–4^). Therefore, regular and long-term monitoring of the heavy metal content of fish in this fishery is recommended.

## Data Availability

The datasets used and/or analyzed during the current study are available from the corresponding author on reasonable request.
